# Antifreeze Protein Supplementation During the Warming of Vitrified Bovine Ovarian Tissue Can Improve the Ovarian Tissue Quality After Xenotransplantation

**DOI:** 10.3389/fendo.2021.672619

**Published:** 2021-05-28

**Authors:** Hyun Sun Kong, Yeon Hee Hong, Jaewang Lee, Hye Won Youm, Jung Ryeol Lee, Chang Suk Suh, Seok Hyun Kim

**Affiliations:** ^1^ Department of Obstetrics and Gynecology, Seoul National University Bundang Hospital, Seongnam, South Korea; ^2^ Department of Obstetrics and Gynecology, Seoul National University College of Medicine, Seoul, South Korea; ^3^ Department of Biomedical Laboratory Sciences, Eulji University, Seongnam, South Korea

**Keywords:** antifreeze protein, ovarian tissue, xenotransplantation, vitrification, warming

## Abstract

The occurrence of ice crystallization during ovarian tissue (OT) cryopreservation causes unavoidable cryodamage, and ice recrystallization during the warming is more detrimental than ice crystallization. Here, we investigated that antifreeze protein (AFP) treatment during the warming procedure can improve the bovine OT quality after xenotransplantation (XT). Bovine OTs (n=120) were evenly assigned to four groups: fresh, vitrified-warmed, vitrified-warmed with 10 mg/mL Leucosporidium ice-binding protein (LeIBP, a type of AFP) (LeIBP-10), and vitrified-warmed with 20 mg/mL LeIBP (LeiBP-20). LeIBPs were added to the first warming solution. Twenty pieces of OTs were assigned to each category. The remaining 10 OTs from each category were assigned to the XT-Fresh control, XT-Vitrified-warmed control, XT-LeIBP-10, and XT-LeIBP-20 groups, respectively, and xenotransplanted to 9-week-old ovariectomized nude mice for one week. LeIBP treatment during the warming step increased morphological follicle normality and decreased apoptotic follicle ratios after vitrification-warming and XT. The XT-vitrified-warmed control group showed significantly reduced microvessel density and increased fibrosis when compared to that of the XT-fresh group. Microvessel density and fibrosis were recovered in both LeIBP treated groups. There was no significant difference between the LeIBP-10 and LeIBP-20 groups in all outcomes. AFP treatment during the warming procedure can prevent OT damage, and improve ovarian follicle morphology and apoptosis in both the vitrified-warmed bovine OT and its graft. After confirmation in a human study, AFPs can potentially be applied to human OT cryopreservation to reduce cryodamage and improve the OT quality.

## Introduction

Ovarian tissue (OT) cryopreservation has been used for young female cancer patients (1). Unlike other fertility preservation options like oocyte and embryo cryopreservation, OT cryopreservation can be applied to a prepubertal girl who cannot receive controlled ovarian hyperstimulation, a woman who does not have a partner, or if chemotherapy cannot be delayed (2, 3). A few weeks after OT transplantation, the function of OT grafts is recovered, and natural pregnancy can be achieved when the OTs are transplanted near or at their original sites (4).

Despite many advantages, OT cryopreservation requires additional improvements to overcome concomitant problems, including cryodamage. Ice crystal formation occurs during the cryopreservation and warming processes, which induces mechanical and osmotic damage in the cryopreserved OT cells (5, 6), and decreases the quality and survival of the OT after thawing and transplantation (7).

Antifreeze proteins (AFPs) or ice-binding proteins are known to help organisms survive in subzero temperatures by lowering the freezing point below the melting point and/or by inhibiting ice crystallization and recrystallization (8–11). Previous studies have demonstrated the beneficial effect of AFPs in preventing cryodamage during the cryopreservation of cells and tissues (12–19). Several studies also have confirmed the cryoprotective effects of AFPs in mouse models with regards to OT cryopreservation (16, 17). However, AFP treatment in large animal models has not been well studied.

Mouse OTs differ from human OTs in various features including size, texture, and follicle density. Thus, studies on OT cryopreservation using AFPs need to be performed in large animal models to obtain a better understanding of the clinical application of AFPs. Bovine OTs have features similar to human OTs in many aspects, including the size and texture of OTs, and the morphology, density, and distribution of ovarian follicles (20, 21). Therefore, bovine OTs have been used for many OT cryopreservation studies (22, 23).

The optimal AFP treatment for bovine OT cryopreservation has not yet been established. It has been demonstrated that 10 mg/mL of Leucosporidium ice-binding protein (LeIBP), a type of AFP, had beneficial effects on the quality of vitrified-warmed mouse OTs (16). In addition, our previous study indicated that adding LeIBP only to the first step of warming solution improved the OT quality comparable to supplying LeIBP in both vitrification and warming solutions (24). To investigate the effects of AFP on vitrified bovine OT, we added 10 and 20 mg/mL of LeIBP during the first step of the OT warming procedure. Furthermore, the warmed bovine OTs were xenotransplanted into nude mice to evaluate the long-term, and ultimate effects of AFP on OT quality. The purpose of this study was to demonstrate the cryoprotective effect of AFP (LeIBP) on vitrified OT warming and transplantation by treating with AFPs only in the warming process in the bovine model.

## Materials and Methods

### Study Design

**Figure 1:**
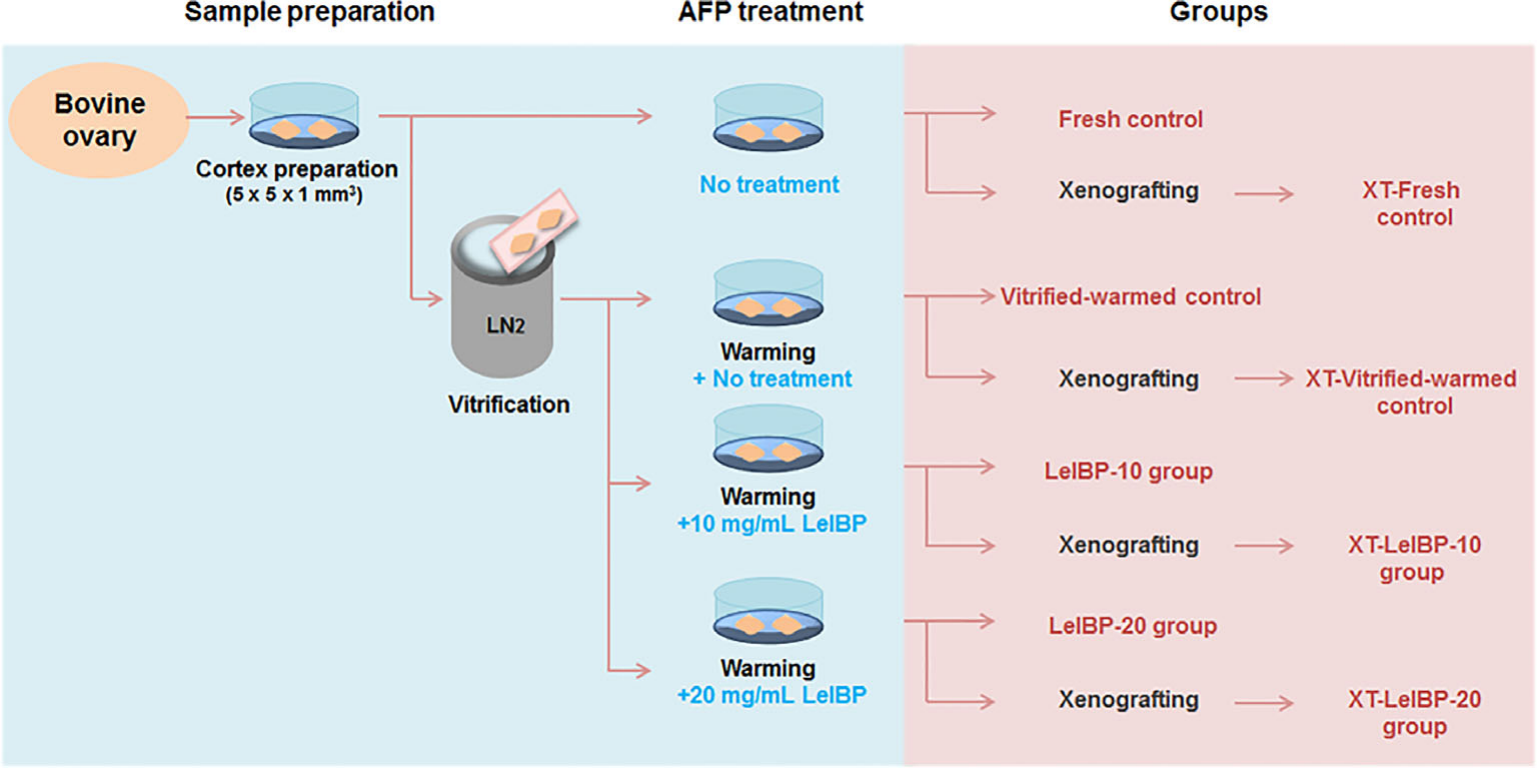
Experimental scheme of this study. Bovine ovarian tissues were prepared as fresh or three different groups of vitrified-warmed tissues in accordance with antifreeze protein (AFP) treatment conditions. The ovarian tissues were either immediately fixed or xenografted for further evaluation. The immediately fixed tissues were classified again as Fresh control, Vitrified-warmed control, LeIBP-10 and LeIBP-20 groups. The xenografted tissues were divided into XT-Fresh control, XT-Vitrified-warmed control, XT-LeIBP-10 and XT-LeIBP-20 groups.

Bovine OTs (n=120) were randomly divided into four different categories depending on whether they were vitrified, and whether AFP (10 mg/mL LeIBP and 20 mg/mL LeIBP) was supplemented to the warming solution, as indicated in **Figure 1**. LeIBP was provided by other researchers who isolated LeIBP from Arctic yeast Leucosporidium sp. AY30 and cloned. The detailed information on LeIBP production was described in their previous report (25).

From each of the four categories, OTs (n=80, 20/group) were again assigned to the fresh control (not vitrified and warmed), vitrified-warmed control (vitrified and warmed without LeIBP), LeIBP-10 (vitrified without LeIBP and warmed with 10 mg/mL LeIBP), and LeIBP-20 (vitrified without LeIBP and warmed with 20 mg/mL LeIBP) groups, respectively.

The remaining OTs (n=40, 10/group) from each category were not immediately analyzed, but were used for subsequent xenotransplantation (XT) as the group assignment: XT-Fresh control, XT-Vitrified-warmed control, XT-LeIBP-10, and XT-LeIBP-20 groups, respectively. For the XT group, the graft and serum samples were collected and analyzed one week after the XT.

### Preparation of Bovine OT

Bovine ovaries were transported from a local slaughterhouse to the laboratory within 2 h. After washing the ovaries, the cortex was separated from the ovarian medulla and cut into 5 × 5 × 1 mm^3^ sized OT pieces. Some of the fresh OTs were immediately fixed in Bouin’s solution (Sigma-Aldrich, St. Louis, MO, US) for morphological evaluation or were xenografted for further evaluation. The remaining OTs were used for subsequent vitrification-warming procedures.

The bovine OTs were vitrified using a slightly modified two-step vitrification method (26). Briefly, OTs were immersed in 7.5% ethylene glycol (EG; Sigma-Aldrich) and 7.5% dimethyl sulfoxide (DMSO; Sigma-Aldrich) in L-15 medium with 20% fetal bovine serum (FBS; Gibco, Waltham, MA, USA) for 15 min at room temperature (RT). The OTs were submerged in the second step solution containing 20% EG, 20% DMSO, and 0.5 M sucrose (Sigma-Aldrich) in L-15 medium with 20% FBS for 10 min at RT. After that, the OTs were placed on a vitrification device (Cryotissue^®^; Kitazato BioPharma, Shizuoka, Japan). The device was directly plunged into liquid nitrogen (LN_2_) and stored in the LN_2_ tank for one day.

The OTs were processed in serially diluted four-step warming solutions containing 1 M, 0.5 M, 0.25 M, and 0 M of sucrose in L-15 medium with 20% FBS. The OTs were immersed in the first step-warming solution for 1 min at 37°C. After that, the OTs were transferred to the successive step-warming solutions for 5 min each at RT. For the LeIBP treatment groups, 10 or 20 mg/mL of LeIBP was added to the first step of warming solution. In accordance with the groups, the vitrified-warmed OTs were fixed immediately or xenografted for further evaluation.

### Xenotransplantation Into Nude Mice

Nine-week-old BALB/c nude mice (Orient Co., Seoul, South Korea), housed under a 12-hour light/dark cycle at 22°C and fed *ad libitum*, were used in the study. All experimental procedures were approved by the Institutional Animal Care and Use Committee (IACUC) of the Seoul National University Bundang Hospital (Approval number: BA1707-227/063–01). The nude mice (n=20, 5/group) were anesthetized with isoflurane gas (4–5% for induction, 1–2% for maintenance).

After anesthesia, the bilateral dorsal part of the mice was opened for ovariectomy. Through the opened hole, bovine OTs were subcutaneously xenografted. After the xenografting procedure, gentamicin (0.75 mg/mouse) was injected intraperitoneally into the mice.

### Collection of Ovarian Samples From the Recipient Mice

The mice were sacrificed one week after XT, for blood and graft collection. After anesthesia, the blood was collected by cardiac puncture. The OT grafts were then retrieved from the subcutaneous site, and the residual mice origin tissues were eliminated. The grafts were washed with normal saline and immediately fixed in Bouin’s solution (Sigma-Aldrich) for one day, followed by embedding in paraffin blocks for histological analysis.

### Histological Analysis

The paraffin blocks embedded with OTs and OT grafts were serially sectioned at 5 μm-thickness. Hematoxylin (DAKO, Seoul, Korea) and eosin (Merck, Darmstadt, Germany) staining was performed at intervals of 100 μm. The remaining slides were used for further analysis.

The H&E slides were blindly read by a single experienced inspector to evaluate the follicular development stages and morphological normality using a light microscope (Nikon, Tokyo, Japan). The developmental stages of follicles were determined in accordance with the following categories (27): (1) primordial follicles: a single layer of flattened pregranulosa cells; (2) primary follicles: a single layer of cuboidal granulosa cells; (3) secondary follicles: two or more layers of cuboidal granulosa cells with the antrum absent; (4) antral follicles: multiple layers of cuboidal granulosa cells with the antrum present.

Morphological evaluation of follicles was performed according to the following criteria. Since the 1 mm-thick ovarian cortical tissue mainly contains primordial follicles, the follicles were classified into two stages as primordial and growing follicles instead of four stages as primordial, primary, secondary, and antral follicles. Only follicles with clearly visible oocytes encircled by granulosa cell layer(s) were counted. Follicles were considered degenerated if they had pyknotic bodies within the granulosa cells, condensed oocyte nuclei, shrunken oocytes, oocyte cytoplasm vacuolization, or low cellular density (28). Otherwise, follicles were classified as morphologically normal.

For follicle density measurement of OT grafts, the H&E stained slides were scanned with a Leica slide scanner (Leica Biosystems, Wetzlar, Germany) at ×200 magnification. Using the scanned images, the graft areas were measured as described in a previous study (29).

### Analysis of Follicular Apoptosis

Apoptosis in the ovarian follicles was detected using terminal deoxynucleotidyl transferase dUTP nick-end labeling (TUNEL) assay using the DeadEnd™ Colorimetric TUNEL System (Promega, Madison, WI, USA) according to the manufacturer’s protocol. Cells with fragmented DNA were visualized as brown with diaminobenzidine, and counterstained with hematoxylin. Follicles containing more than 30% apoptosis-positive cells were defined as apoptotic follicles (26).

### Evaluation of the Microvessels in the OT Graft

CD31 immunostaining was performed to observe the microvessels in the OT grafts. Paraffin slides were deparaffinized and rehydrated in xylene and ethanol, respectively. Target antigen retrieval of the ovarian graft was performed, followed by peroxidase blocking. The slides were then treated with CD31 antibody (1:400, Bioss, Woburn, MA, USA) for 1h at RT. After that, the slides were washed and treated with EnVision/HRP solution (DAKO) for 30 min, and substrate-chromogen solution (DAKO) for 10 min followed by counterstaining with hematoxylin. The slides were scanned with a Leica slide scanner at ×200 magnification, and image analysis was performed using the i-Solution image analysis software (IMT i-Solution Inc., Daejeon, Korea).

### Measurement of Graft Fibrosis

Masson’s trichrome staining was performed to measure fibrosis in the OT graft using the Roche Trichrome III Blue Staining Kit (Roche, Basel, Switzerland). The fibrotic surface, nuclei, and cytoplasm space were stained as blue, black, and red, respectively. The stained slides were scanned with a Leica slide scanner at × 200 magnification, and analyzed using the i-Solution image analysis software for fibrotic surface area analysis.

### Hormonal Assay

Serum was separated from the blood sample of each recipient mouse by centrifugation at 3,000 × g and 4°C, for 10 min. The serum was stored at −80°C, and used for enzyme-linked immunosorbent assay (Cusabio, Wuhan, China) of bovine estradiol.

### Statistical Analysis

The results of follicular normality and apoptosis were analyzed by chi-square test using the statistical software package SPSS 18.0 (SPSS Inc., Chicago, IL, USA). One-way analysis of variance (ANOVA) was used to analyze the follicle density of the ovarian graft, and the Kruskal-Wallis test was used to measure graft microvessels, fibrosis and hormonal assays, both using the statistical analysis software GraphPad Prism version 6.0 (Graph-Pad, San Diego, CA, USA). The outcomes were considered as significantly different when the p value was less than 0.05.

## Results

### Evaluation of Ovarian Follicle Morphology and Apoptosis After Vitrification-Warming

A total of 7,317 follicles were evaluated of their morphological normality, as shown in **Table 1**. Regardless of follicle developmental stage, the morphologically normal follicle ratios were significantly lower in the vitrified-warmed control (primordial follicle: 65.6%, growing follicle: 46.3%, total follicle: 57.6%) than in the fresh control (primordial follicle: 80.1%, growing follicle: 64.4%, total follicle: 74.6%). However, the ratios were significantly improved in both LeIBP treated groups (LeIBP-10: primordial follicle: 76.8%, growing follicle: 58.4%, total follicle: 70.0%; LeIBP-20: primordial follicle: 75.4%, growing follicle: 57.8%, total follicle: 67.7%) compared to that in the vitrified-warmed control.

**Table 1:** Proportions of morphologically normal and apoptotic follicles in four different groups after vitrification-warming.

Groups	Ovarian tissue No.	Follicular normality			Apoptotic follicle
		Primordial	Growing	Total	
Fresh	20	80.1% (979/1222)^a^	64.4% (433/672)^a^	74.6% (1412/1894)^a^	17.2% (83/482)^a^
Vitrified-warmed	20	65.6% (586/893)^b^	46.3% (298/643)^b^	57.6% (884/1536)^b^	31.8% (148/466)^b^
LeIBP-10	20	76.8% (940/1224)^c^	58.4% (420/719)^c^	70.0% (1360/1943)^c^	23.9% (113/472)^c^
LeIBP-20	20	75.4% (826/1096)^c^	57.8% (490/848)^c^	67.7% (1316/1944)^c^	23.6% (109/462)^c^

•The results of follicular normality and apoptosis were analyzed by chi-square test.

•Different superscript means a statistical significance within the same column.

The apoptotic follicle ratios of both LeIBP treated groups (LeIBP-10: 23.9%, LeIBP-20: 23.6%) were significantly reduced when compared to that in the vitrified-warmed control group (31.8%), however, it was still higher than that of the fresh control group (17.2%), as indicated in **Table 1**.

### Evaluation of Ovarian Follicle Morphology and Apoptosis in the Xenotransplanted Ovarian Grafts

**Figure 2:**
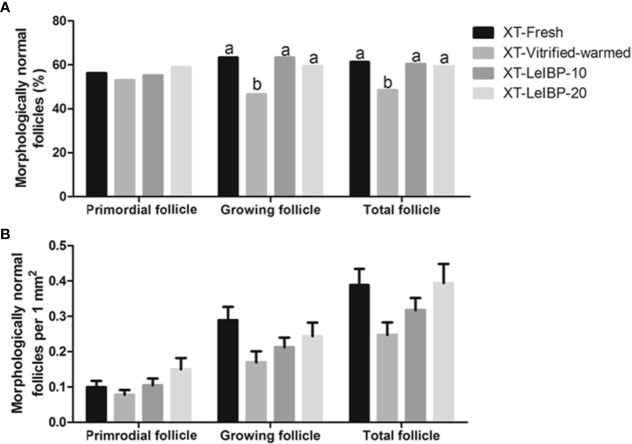
Proportions and densities of morphologically normal follicles according to the different xenografted groups. **(A)** Morphologically normal follicle ratios; **(B)** morphologically normal follicle densities per 1 mm^2^ of ovarian grafts. Different superscript letters indicate statistically significant differences (P < 0.05). XT, xenotransplantation.

In terms of primordial follicle normality, there was no significant difference among all groups, as shown in **Figure 2A**. In the case of growing follicles, the follicle normality was significantly decreased in the XT-Vitrified-warmed control group (46.6%) compared to that in the XT-Fresh control group (63.3%), and improved in the XT-LeIBP-10 and XT-LeIBP-20 groups (63.3% and 59.5%, respectively) with a remarkable difference. These results were similar to those of the total follicle normality ratios (XT-Fresh: 61.3%, XT-Vitrified-warmed: 48.4%, XT-LeIBP-10: 60.4%, XT-LeIBP-20: 59.3%).

Morphologically normal follicle densities were measured per 1 mm^2^, and the results are illustrated in **Figure 2B**. Increasing trend was observed in LeIBP treated groups in all follicle developmental stages, but no significant difference was found among the groups.

**Figure 3:**
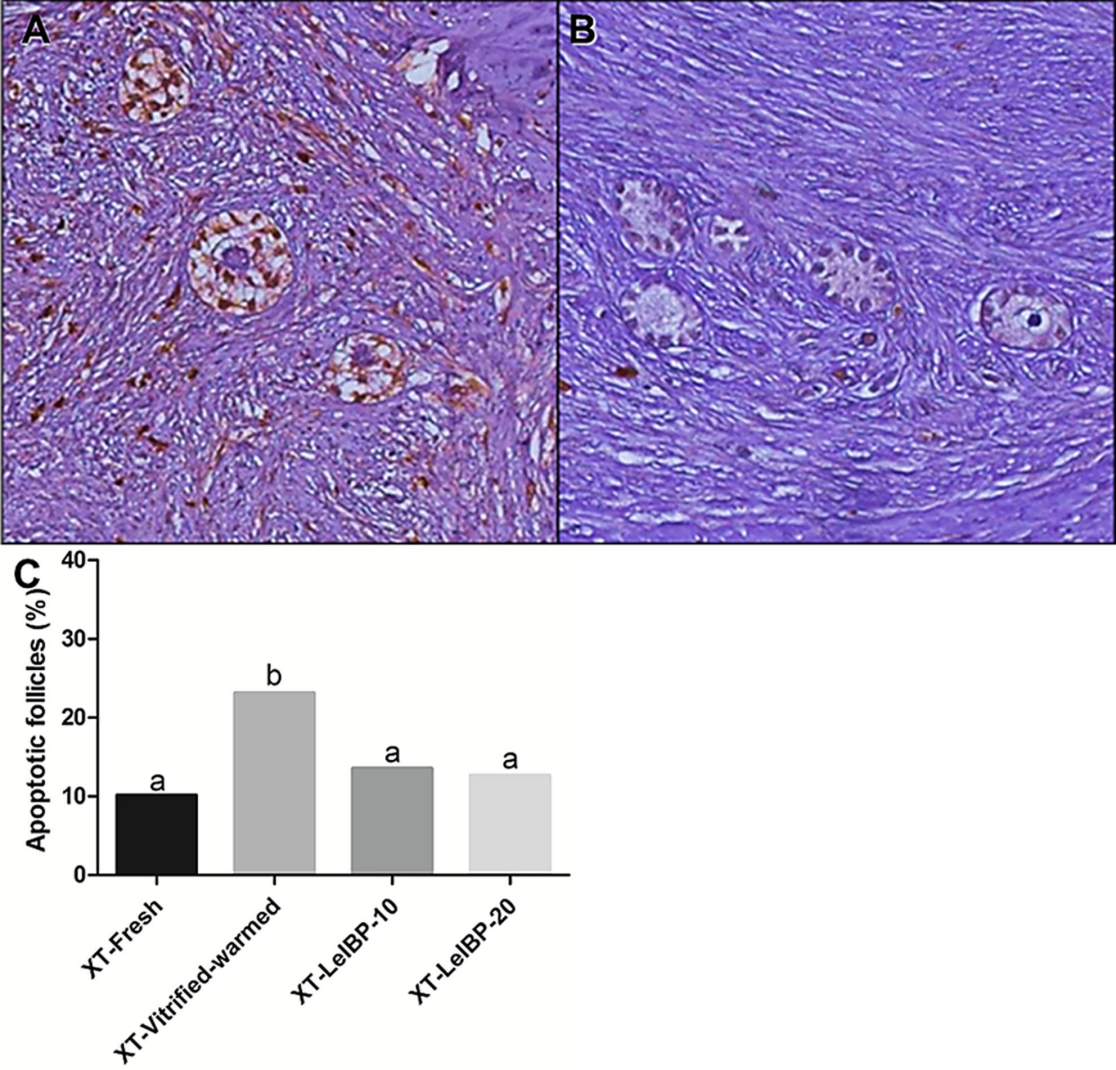
Apoptotic follicle evaluation of ovarian grafts. Each panel indicates **(A)** TUNEL positive follicles (×200 magnification); **(B)** TUNEL negative follicles (×200 magnification); **(C)** apoptotic follicle ratio graph according to the different xenografted groups. Different superscript letters indicate statistically significant differences (P < 0.05). TUNEL, terminal deoxynucleotidyltransferase-mediated dUTP nick end labeling; XT, xenotransplantation.

The apoptotic follicle ratios after XT are indicated in **Figure 3**. Compared to the XT-Vitrified-warmed control (23.1%), apoptotic follicle ratios significantly decreased in the fresh and LeIBP treatment groups (XT-Fresh: 10.1%, XT-LeIBP-10: 13.5%, LeIBP-20: 12.6%).

### Microvascularity and Fibrosis of Xenotransplanted Grafts

**Figure 4:**
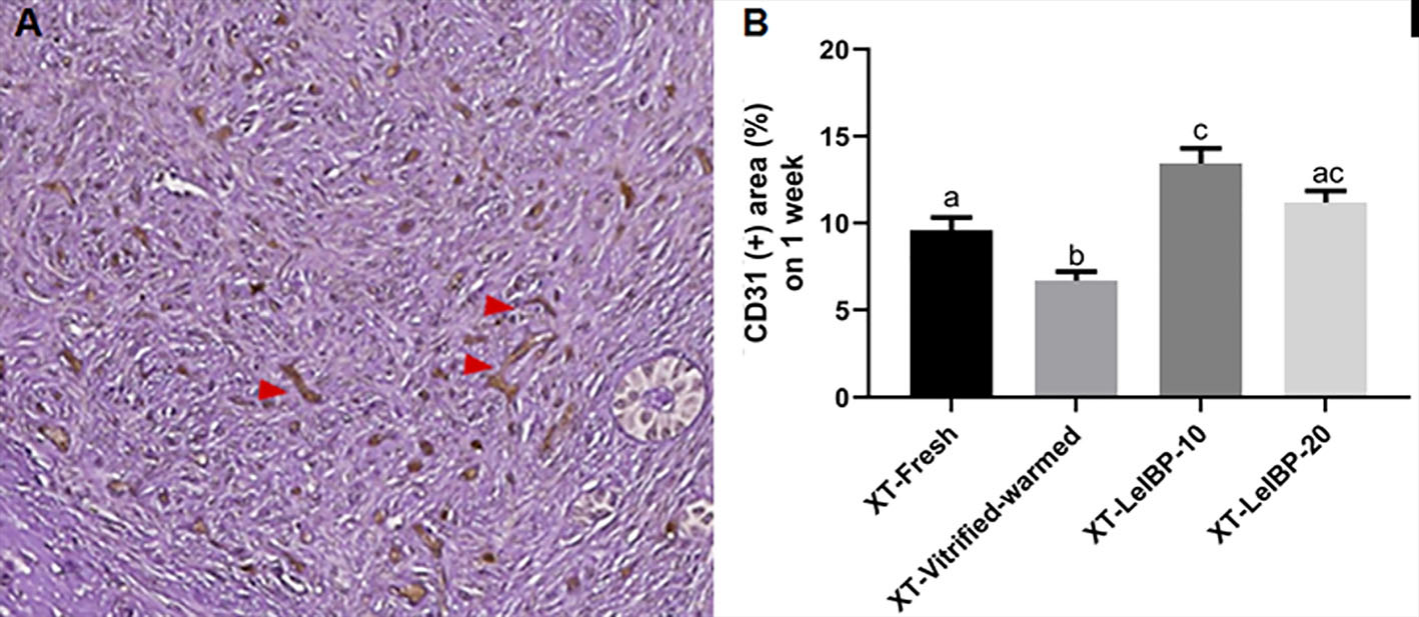
Microvessel assessment of ovarian grafts. **(A)** A representative image of xenografted ovarian graft immune-stained for CD31. The red arrowheads indicate CD31 positive microvessels stained with the brown color (× 200 magnification). **(B)** Ratios of CD31 positive area according to the different xenografted groups. Different superscript letters indicate statistically significant differences (P < 0.05). XT, xenotransplantation.

A representative figure of CD-31 immunostained OT is given in **Figure 4A**. Among the four groups, the proportion of CD31-positive area was lowest in the XT-Vitrified-warmed control group (6.7 ± 0.5%), and was significantly increased in the XT-LeIBP-10 (13.4 ± 0.9%) and XT-LeIBP-20 (11.2 ± 0.7%) groups (**Figure 4B**).

**Figure 5:**
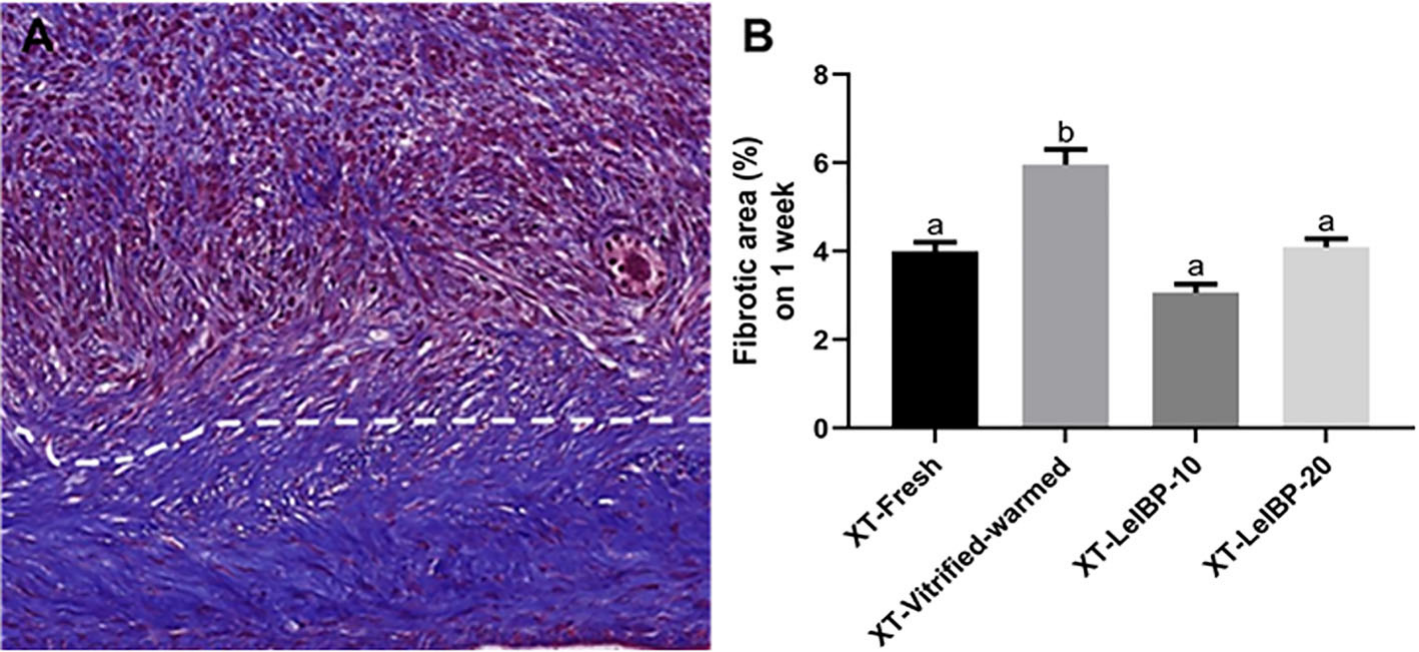
Fibrotic area assessment of ovarian grafts. **(A)** A representative image of Masson’s trichrome stained ovarian graft. The upper part of the white dotted line indicates less fibrotic graft while the lower part indicates extensive fibrotic area. The fibrotic surface, nuclei, and cytoplasm were stained blue, black, and red, respectively (× 200 magnification). **(B)** The fibrotic area ratios according to the different xenografted groups. Different superscript letters indicate statistically significant differences (P < 0.05). XT, xenotransplantation.

A representative image of the Masson’s trichrome-stained graft is given in **Figure 5A**. The upper part of the white dotted line indicates the less fibrotic area, whereas the lower part indicates the extensive fibrotic area (× 200 magnification). The fibrotic area proportion was highest in the XT-Vitrified-warmed control group (6.0 ± 0.3%), and was significantly reduced in the LeIBP treatment groups (XT-Fresh: 4.0 ± 0.2%; XT-LeIBP-10: 3.1 ± 0.2%; XT-LeIBP-20: 4.1 ± 0.2%) (**Figure 5B**).

### Serum Estradiol Levels After XT of Bovine OT

**Figure 6:**
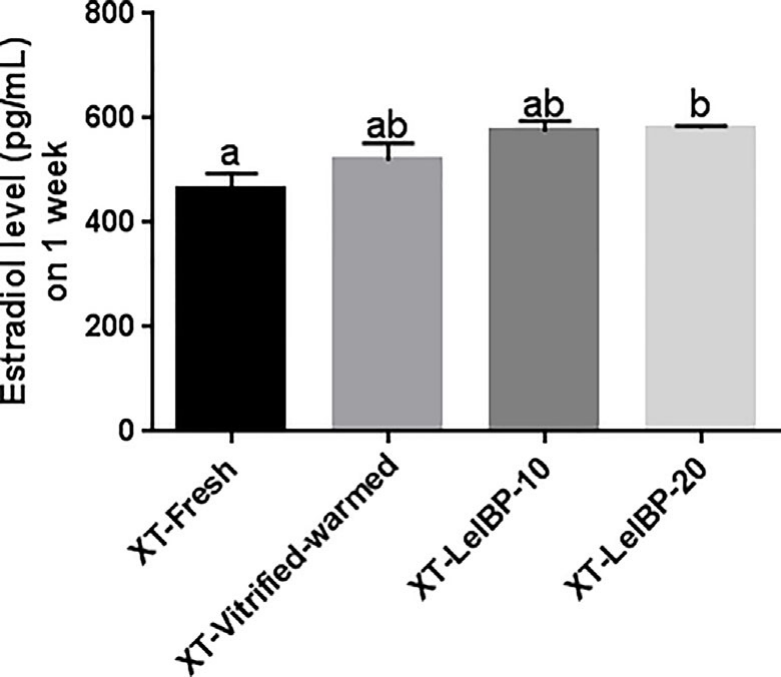
Measurement of serum estradiol levels. The serum estradiol level is presented according to the different xenografted groups. Different superscript letters indicate statistically significant differences (P< 0.05). XT, xenotransplantation.

Serum estradiol was detected one week after the XT. The XT-LeIBP-20 group demonstrated significantly higher serum estradiol levels compared to that in the XT-Fresh control group (**Figure 6**). There was no significant difference among the other groups.

## Discussion

In the present study, the bovine OTs were treated with 10 mg/mL or 20 mg/mL LeIBP during the first step of the OT warming process to demonstrate the effects of AFP on recrystallization. The bovine OTs were xenotransplanted into nude mice to evaluate the subsequent and prolonged effects of AFP on OT survival and quality. A significant improvement in follicle morphologic normality was observed in the LeIBP-treated groups when compared with the vitified-warmed control group. In accordance with our previous study using a mouse model (24), LeIBP treatment during the warming step showed improved outcomes in the bovine OT. There was no significant difference between the 10 mg/mL LeIBP and 20 mg/mL LeIBP groups in terms of morphological normality, which did not show a dose-dependent increase. Based on these data, we demonstrate that AFP (LeIBP) treatment of bovine OT during the warming process improved the quality of OTs and OT grafts.

Cryodamage, such as mechanical and/or osmotic damage caused by ice crystal formation, can induce apoptosis in cells (5, 6). Previous studies have demonstrated that AFP treatment reduces apoptosis in cells and/or tissues at subzero conditions (12, 16, 17, 24). In the present study, the LeIBP treated groups demonstrated significantly decreased apoptotic follicle ratios than the Vitrified-warmed control group. The use of different LeIBPs doses (10 mg/mL and 20 mg/mL of LeIBP) did not have significantly different anti-apoptotic effects on cryopreserved bovine OTs, which is similar to our previous study using mice (24). Therefore, we suggest that 10 mg/mL of LeIBP is a sufficient and effective concentration for bovine OT cryopreservation.

In the OT xenotransplantation study, the normality of growing follicles and total follicles significantly improved in the XT-LeIBP-10 and -20 groups when compared to that in the XT-Vitrified-warmed control group. However, no significant difference in the density of morphologically normal follicles was found among all XT groups, although both LeIBP treated groups showed a non-significant increasing trend of follicle density with the dose increase. The reason for this result in follicle density may be due to the small sample size of follicle number.

The apoptotic follicle ratio was highest in the XT-Vitrified-warmed control group when compared to that of the other groups. Both treatment groups showed significantly reduced apoptotic follicle ratios. The results of the vitrified-warmed and non-XT OT groups showed similar patterns. This suggests that cryodamage affects OT quality even after transplantation. LeIBP treatment reduced cryodamage in OTs, and displayed reduction in follicle apoptosis even after the XT procedure.

In our study, all OT grafts in the 4 groups suffered ischemic injury due to XT, as also demonstrated by several studies showing decreased microvessel counts in vitrified-warmed OT grafts when compared to that in fresh OT grafts (7, 30). Our XT-Vitrified-warmed control group displayed a significantly lower proportion of CD31(+) microvessel area compared to that in the XT-Fresh control and LeIBP treated XT groups, indicating that AFP affects neovascularization of OT grafts by improving the quality of vitrified-warmed OT. These results are consistent with those of our previous study, which examined vascularization during OT vitrification and transplantation in mouse ovaries (7). On comparing the CD31(+) area in the fresh OT without transplantation and in the vitrified ones, a significant decrease in the area in the latter was observed. This trend persisted until after OT transplantation, and the pattern was consistently observed during subsequent periods (Day 2, Day 7, Day 21), with the results showing a significant decrease, especially on day 21. Therefore, in the present study, the CD31(+) area in the LeiBP added groups was significantly increased compared to that in the group without it, regardless of the LeiBP concentration. As indicated by this result, the addition of LeiBP during the warming process might have a positive effect on the vitrified–warmed tissues by reducing the cryodamage and increasing vessel density after transplantation.

Several studies have reported that ischemic injury leads to extensive fibrosis of the OT grafts, demonstrating the close correlation between ischemic damage and graft fibrosis (27, 31). In this study, the fibrotic area ratios were significantly decreased in the XT-LeIBP-10 and -20 groups when compared to those in the XT-Vitrified-warmed control group, indicating that the LeIBP-treated OT grafts had high microvessel counts, low fibrosis, and subsequently low ischemic injury.

The fundamental cause of decreased cell viability during cryopreservation is ice recrystallization (IR). Previous studies that used freezing medium containing LeIBP have shown better post-thaw viability compared to that of other marine-derived AFPs (16, 32, 33). Kong et al. demonstrated that the addition of LeiBP in the first step of the warming procedure could reduce follicle apoptosis to the same degree as that achieved with the conventional protocol using LeiBP in both vitrification and warming solutions in mouse OT transplantation (24). Likewise, alleviating cryoinjury generated by IR can improve cell survival in both ovarian follicles and endothelial cells (ECs) surrounding the follicles in the bovine tissue transplant, when LeiBP was added during the warming step. Since ECs may elaborate essential angiocrine factors involved in organ regeneration (34), the protection of endothelial cells from cryodamage could be one way to improve the efficacy of post-thaw OT transplantation. From this point of view, LeiBP is a promising protein for tissue cryoprotection. In addition to the ice recrystallization inhibition (IRI) feature, the interaction of LeiBP with membranes or membrane proteins may ameliorate cryoinjury in cells (35). Protection against cryodamage during warming with LeiBP treatment reduces ischemic-reperfusion injury and fibrosis by increasing vessel density after OT transplantation. The same results were also observed in previous papers on mouse OT transplantation (16, 24). Another possible role of LeiBP is antioxidant activity. Zheng et al. showed that antioxidants have a cryoprotective effect (36), and the cryoprotective effect of LeiBP in sperm freezing is closely related to antioxidant activity, showing increased superoxide dismutase activity (37). Similarly, OT is inevitably exposed to reactive oxygen species (ROS) during the cryopreservation process, therefore, it can be assumed that the added LeiBP may exert an antioxidant effect.

While normal morphology, the degree of apoptosis, or fibrosis is an essential criterion for evaluating the effect of specific molecules, it does not entirely correspond to the viability or function of ovaries. It is necessary to measure the establishment of follicle activity to evaluate the effectiveness of the cryopreservation protocol. Serum estradiol levels were measured to investigate whether the xenografted OTs were well settled in the XT site and functioning. Donor mice in all XT groups secreted bovine estradiol, demonstrating that the OT grafts were well settled and functioned in donor mice. In particular, the XT-LeIBP-20 group showed significantly increased serum estradiol levels compared to the XT-Fresh control. Since we xenografted the ovarian cortex, which includes mostly primordial follicles, evaluation of serum hormone levels only one week after XT seemed inappropriate for accurate hormonal assessment. Additionally, we did not inject hormones to donor mice for cycle synchronization, nor perform vaginal cytology to evaluate the estrous cycle, and evaluated a small number of serum samples (five per group). Therefore, the statistics were considered to have little meaning other than to ensure that the xenotransplanted OTs were well established and functional. We transplanted bovine OT in the dorsal subcutaneous area of the recipient mice, which is known to be a place with poor endocrine function among heterotopic sites due to inadequate blood supply (38). Therefore, an insufficient or unstable function of the ovarian graft could not make a significant difference. For heterotopic transplantation, an effective endocrine function can be achieved when transplanted into a kidney capsule or abdominal muscle rich in blood vessels, and significant differences may be seen when OTs are transplanted in these areas.

Currently, slow freezing is regarded as a standard method of human OT cryopreservation although vitrification is also actively performed in many centers. Though this study was based on vitrified-warmed OT, AFPs were only used in the warming process. The difference in the warming method between slow freezing and vitrification was not significant. Like vitrification, recrystallization during thawing is the leading cause of damage in slow freezing. Therefore, the present study results may also be applicable to OT cryopreservation through slow freezing and thawing.

This study has several limitations. First, we did not observe the long-term effect of LeiBP as the changes were confirmed on the 7th day of OT transplantation. Second, changes in hypoxia-related or ROS-related genes, before and after OT transplantation were not evaluated. Lastly, although we conducted this study in large animals, the efficacy of LeiBP in human OT cryopreservation and OT transplantation has not yet been confirmed. Therefore, further studies on the safety, and elaboration of the protocol are needed in the future.

In conclusion, this is the first report that uses AFP for only the OT warming step following OT vitrification in the bovine model. We demonstrate that AFP has cryoprotective effects on preserving follicle morphology, decreasing follicle apoptosis, improving graft microvessel formation, and reducing graft fibrosis even after transplantation. Our findings will provide the basis for further studies on the mechanism and function of AFPs in human OT preservation. Further studies are necessary to optimize the AFP treatment protocol for application to human OT.

## Data Availability Statement

The raw data supporting the conclusions of this article will be made available by the authors, without undue reservation.

## Ethics Statement

The animal study was reviewed and approved by The Institutional Animal Care and Use Committee (IACUC) of the Seoul National University Bundang Hospital (Approval number: BA1707-227/063–01).

## Author Contributions

HK: Conception and design, experiment, data analysis, data interpretation, drafting, and revision of the article. YH: Conception and design, data interpretation, drafting, and revision of the article. JL: Conception and design, data interpretation, and revision of the article. HY: Conception and design, data analysis, data interpretation, and revision of the article. JL: Conception and design, data analysis, data interpretation, revision and final approval of the article. CS: Conception and design, data interpretation, and revision of the article. SK: Conception and design, data interpretation, and revision of the article. All authors contributed to the article and approved the submitted version.

## Funding

This research was supported by a grant of the Korea Health Technology R&D Project through the Korea Health Industry Development Institute (KHIDI), funded by the Ministry of Health & Welfare, Republic of Korea (grant number: HI18C0081 and HI18C1999).

## Conflict of Interest

The authors declare that the research was conducted in the absence of any commercial or financial relationships that could be construed as a potential conflict of interest.
